# Potential neuroimaging biomarkers of pathologic brain changes in Mild Cognitive Impairment and Alzheimer’s disease: a systematic review

**DOI:** 10.1186/s12877-016-0281-7

**Published:** 2016-05-16

**Authors:** Qingwei Ruan, Grazia D’Onofrio, Daniele Sancarlo, Zhijun Bao, Antonio Greco, Zhuowei Yu

**Affiliations:** Shanghai Institute of Geriatrics and Gerontology, Shanghai Key Laboratory of Clinical Geriatrics, Department of Geriatrics, Huadong Hospital, and Research Center of Aging and Medicine, Shanghai Medical College, Fudan University, Shanghai, 200040 China; Geriatric Unit & Laboratory of Gerontology and Geriatrics, Department of Medical Sciences, IRCCS “Casa Sollievo della Sofferenza”, San Giovanni Rotondo, Foggia Italy; Huadong Hospital, Shanghai Medical College, Fudan University, 221 West Yan An Road, Shanghai, 200040 P.R. China

**Keywords:** Alzheimer’s disease, Mild cognitive impairment, Magnetic resonance imaging, Positron emission tomography

## Abstract

**Background:**

Neuroimaging-biomarkers of Mild Cognitive Impairment (MCI) allow an early diagnosis in preclinical stages of Alzheimer’s disease (AD). The goal in this paper was to review of biomarkers for Mild Cognitive Impairment (MCI) and Alzheimer’s disease (AD), with emphasis on neuroimaging biomarkers.

**Methods:**

A systematic review was conducted from existing literature that draws on markers and evidence for new measurement techniques of neuroimaging in AD, MCI and non-demented subjects. Selection criteria included: 1) age ≥ 60 years; 2) diagnosis of AD according to NIAAA criteria, 3) diagnosis of MCI according to NIAAA criteria with a confirmed progression to AD assessed by clinical follow-up, and 4) acceptable clinical measures of cognitive impairment, disability, quality of life, and global clinical assessments.

**Results:**

Seventy-two articles were included in the review. With the development of new radioligands of neuroimaging, today it is possible to measure different aspects of AD neuropathology, early diagnosis of MCI and AD become probable from preclinical stage of AD to AD dementia and non-AD dementia.

**Conclusions:**

The panel of noninvasive neuroimaging-biomarkers reviewed provides a set methods to measure brain structural and functional pathophysiological changes in vivo, which are closely associated with preclinical AD, MCI and non-AD dementia. The dynamic measures of these imaging biomarkers are used to predict the disease progression in the early stages and improve the assessment of therapeutic efficacy in these diseases in future clinical trials.

## Background

Alzheimer’s disease (AD) is the most common age-related neurodegenerative disease, characterized by progressive cognitive decline, accounts for 50–75 % of the global dementia population, with a greater proportion in the higher age ranges [1].

Clinicians and researchers have recently updated the AD diagnostic criteria for use in clinical practice and research [2]. Biomarkers (e.g., CSF protein levels, neuroimaging) may be used to rule out other causes of dementia (e.g., vascular) and to support the AD diagnosis in cases with unclear or atypical presentations. Attempts to diagnose AD at an earlier stage have led to the appearance of new medical terminologies such as pre-clinical AD, prodromal AD or mild cognitive impairment (MCI). Recently, new criteria for diagnosis of MCI in clinical and research settings have been published [3, 4].

In this context neuroimaging and fluid biomarkers for amyloid deposition and hippocampal atrophy can be measured more than 10 years before the onset of dementia [5, 6]. The application of these markers could enhance the specificity of clinical diagnosis and improve the prediction of the disease progression. The objective in this paper was to review of biomarkers for Mild Cognitive Impairment (MCI) and AD, with emphasis on neuroimaging biomarkers.

## Methods

The search strategy and analysis was informed by: the study’s aims, previous systematic reviews using qualitative data, and best practice recommendations in the research literature [7, 8]. Literature searches were conducted over MEDLINE (2000 to June 2015) and Pubmed (2000 to June 2015), using the OVID search interface. The searches were limited to human studies in English language including potential Magnetic Resonance Imaging (MRI) and Positron Emission Tomography (PET) biomarkers.

### Study selection

A single reviewer examined the abstracts retrieved by the electronic search in order to identify articles that met the inclusion criteria and to be fully reviewed.

Inclusion criteria: 1) age ≥ 60 years; 2) diagnosis of AD according to the National Institute on Aging-Alzheimer’s Association (NIAAA) criteria [2], 3) diagnosis of MCI according to NIAAA criteria [4] with a confirmed progression to AD assessed by clinical follow-up, and 4) acceptable clinical measures of cognitive impairment, disability, quality of life, and global clinical assessments.

Exclusion criteria: 1) no English editing (as we lacked resources for translation), 2) diagnosis of non-AD dementia, and 3) MCI not progressed in AD.

#### Data extraction

**Fig. 1:**
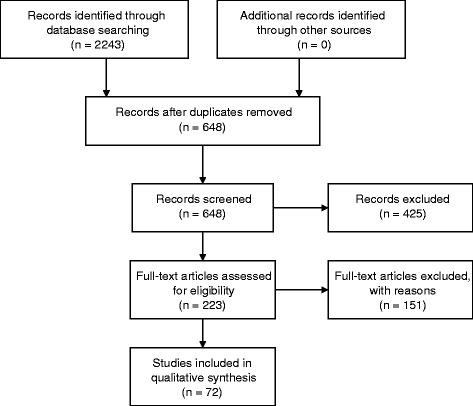
Flow diagram outlining the selection procedure to identify articles which were included in the systematic review of neuroimaging biomarkers of pathologic brain changes in Mild Cognitive Impairment and Alzheimer’s disease

In total, 2243 articles, reports and reviews were identified. After reviewing abstracts, 425 were excluded on the basis of the aforementioned inclusion/exclusion criteria and the removal of duplicates (n. 1595). A further 151 were excluded after more in-depth examination (on the basis of the same inclusion/exclusion criteria). Thus, 72 published studies were eligible for the current review (Fig. 1).

Data extraction followed a number of stages. After preliminary categorisation, categories were divided among the authors according to expertise. Each category was reviewed in depth by at least one author and the lead author:

- Potential structural neuroimaging biomarkers
- Functional neuroimaging biomarkers

Co-authors provided a detailed summary of each study including its strengths and weaknesses, as well as an overall appraisal of the category [9, 10]. Quality of study reporting was assessed using the Standards for the Reporting of Diagnostic accuracy studies in dementia (STARDdem) [11].

## Results and discussions

### Potential structural neuroimaging biomarkers

#### Structural MRI (sMRI)

Medial temporal and hippocampal atrophy were the most common structural MRI (sMRI) markers of progression to AD [12, 13]. The 3-dimensional patterns of cerebral atrophy progression over multiple serial MRI were consistent with neurofibrillary pathological staging scheme in AD, showing that the earliest changes in the anterior medial temporal lobe and fusiform gyrus occur at least 3 years before conversion to AD [14]. However, one study showed that both baseline enthorhinal volume and its slope of decline, but not initial hippocampal size and its rate of decline, were independent predictors of incident AD [15].

Molecular biomarkers of neuronal injury that are present in advance of atrophy offer a complementary target for sMRI [16]. Model-based magnetization transfer (mMT) imaging could improve classification of subjects with early AD and MCI compared with magnetization transfer ratio (MTR) [17].

Individual biomarkers, such as white matter hyperintensities (WMH), cerebral volume, hippocampal volume, entorhinal cortex thickness, ventricle volume, and CSF markers had limited predictive value for cognitive decline [18]. Combination of sMRI and plasma biomarkers, such as whole genome single nucleotide polymorphism data, tocopherols and tocotrienols, or CSF total tau, phosphorylated tau (p-tau) and Aβ42: amyloid-β42 (Aβ42) could enhance the accurancy of differentiating subjects with AD and MCI from cognitively normal subjects [19–21]. Annual ventricular volume changes in serial sMRI were related to concurrent change on general cognitive and functional indices in amnestic MCI (aMCI) and AD, and were influenced by Apolipoprotein E (ApoE) genotype [22]. The combination of imaging and CSF biomarkers could improve the conversion prediction from MCI to AD [21, 23–25] and time to develop dementia in subjects with MCI and amyloid pathology [26]. Baseline fludeoxyglucose (FDG) Positron Emission Tomography (PET) added the greatest prognostic information compared with baseline clinical testing [24], but marginally predicted longitudinal cognitive decline [25]. Baseline sMRI was slightly better predictor of future clinical/functional decline than CSF t-tau/Aβ42 [21]. MRI imaging might be a more practical clinical biomarker for early detection of AD [27]. Multiple MRI markers of underlying dementia pathologies improve the ability to identify patients with prodromal dementia over a single MRI marker [28]. The areas of hypoperfusion in patients with asymmetric cortical degenerative syndromes measured by single-photon emission computed tomography (SPECT) were larger than the corresponding atrophic regions showed by sMRI [29]. The combination of both was useful in identifying the regional structural and functional cerebral abnormalities.

The mild or severe atropy in sMRI with severe decline in parietal regional Cerebral blood flow (CBF) on SPECT could distinguish AD from FTD and VaD patients [30]. The most common non-AD dementia is vascular dementia (VaD) with considerable overlap with AD pathology. VaD results from cerebrovascular and cardiovascular disorders. Non-significant vascular lesions on structre brain imaging result in overdiagnosis of VaD [31]. A significantly higher allele frequency of ApoEε4 in AD patients was found than these with VaD in a community population [32]. The increase of WMH in deep white matter, but not WMH in periventricular white matter on MRI scans indicates 86 % classification accuracy [33]. MRI measure of infarcts, a vascular brain injury, were significantly related to medial-temporal and cerebral atropy in VaD but not in AD patients with 71 % sensitivity and 76 % specificity [34]. The combination of MRI and SPECT could differentiate VaD with sensitivity 88 %, specificity 75 % and FTD with sensitivity 73 %, specificity 78 % respectively, and enhance specificity of AD diagnosis by CSF biomarkers from 71 to 93 % [35].

#### Diffusion MRI

The diffusion tensor imaging (DTI) metrics including fractional anisotropy (FA), mean diffusivity, axial diffusivity and radial diffusivity (RA), can assess the connectivity between brain regions [36–38]. Both vascular and AD degenerative process have different region-specific structural injury patterns of cerebral white matter [39]. The combination of mean tanscallosal prefrontal FA and the Fazekas score in volume by T2-weighted measures could differentiate VaD from AD with 87.5 % accuracy, 100 % specifity and 93 % sensitivity [40].

The reduced FA and RA values in patients with AD than in patients with MCI suggested that the brain DTI was useful to confirm clinical manifestation of AD but not in the detection of MCI [41]. White matter microstructural changes identified with DTI derived FA doesn’t appear as a significant predictor of cognition [42]. In another study, it was shown that high WMH predict progression from normal to MCI, but not progression from MCI to dementia [43]. Conversely, the brain parenchymal fraction (BPF) which showed whole-brain atrophy, did not predict conversion from normal to MCI but predicted conversion to dementia [43]. In a study, it was shown that posterior periventricular and corpus callosum extension of WMH associated with MCI and AD indicate involvement of strategic white matter bundles that may contribute to the cognitive deficits [44]. Increased amyloid burden, as measured with florbetapir PET imaging, was associated with changes in weighted structural connectome metrics independent of brain region [45]. The decrease of FA and the mode of anisotropy in intracortically projecting fiber tracts of MCI-AD and AD subjects suggested early axonal degeneration in intracortical projecting fiber tracts [46]. To assess the decrease of the cingulum fibers using DTI-derived FA could help early diagnosis of AD [47]. The hippocampal apparent diffusion coefficients were higher in MCI and AD subjects than in controls [48]. The elevated apparent diffusion coefficients in hippocampus may indicate early pre-clinical AD.

### Functional neuroimaging biomarkers

#### PET imaging for metabolism status

A study showed that 30 min was enough foroptimal FDG accumulation for AD PET scan, especially for the early stage of AD [49]. Bilateral temporoparietal hypometabolism on [18 F] fludeoxyglucose-positron emission tomography (FDG-PET) could differentiate AD dementia from VaD dementia with leukoaraiosis [50]. (18)FDG-PET showed FDG uptake reductions in AD related brain regions in cognitively normal individual with ApoEε4 genotype and subjective cognitive complaint [51], or a maternal history of AD [52]. Compared with nonconverters, converters of MCI patients had lower FDG uptake in the right temporoparietal contex [53]. Hypometabolism in brain regions may be related to ongoing pathologies and to reduced neuronal input in remote but functionally connected regions [54]. MCI patients who did not develop dementia after 2 years showed even higher uptake in the basal forebrain than those who developed dementia [55]. Longitudinal changes in individual subjects in the spatial pattern of brain glucose metabolism measured with (18)F-FDG PET showed correlations with the cognitive decline of AD and MCI [56, 57].

The combination C-labeled Pittsburgh Compound-B ([(11)C]PIB) and FDG track molecular changes could show different stages of AD. Increased [(11)C]PIB images of binding potential was found in MCI patients and progressive decrease of FDG uptake was only observed in AD patients [58]. The hypoperfusion patterns showed by arterial spin labeling MRI and hypometabolism patterns showed by FDG-PET could provide largely overlapping information of fuctional deficits in affected areas of AD [59]. Regional cerebral blood flow measured by [11C]dihydrotetrabenazine (DTBZ)-PET also provided similar information in assessment of regional cerebral metabolic deficits in mild dementia and MCI with FDG-PET, and exhibited a mild decrease in sensitivity [60]. Preclinical and clinical studies have shown that the use of PET imaging for tracking neuroinflammatory changes seems to have a promising role in AD and other central nervous system (CNS) pathologies [61, 62]. Translocator protein (TSPO) radioligands [11C](R)-PK11195, [11C]DAA1106 and [11C]PBR28 suggest the increased expression of TSPO by activated microglia in AD patients [62].

The radioligand 11C-deuterium-L-deprenyl ([11C]-DED) showed the highest binding among PIB in MCI individuals, that suggests the reactive astrocytosis occurs at the early stages of AD [63]. Activation of cytosolic phospholipase A2 (cPLA2) and secretory phospholipase A2 (sPLA2) after microglia-derived inflammatory cytokines bind astrocytic cytokine receptors results in the hydrolysis of membrane phospholipids, liberating arachidonic acid (AA) [64]. A common single nucleotide polymorphism (rs6971) in exon 4 of the TSPO gene has been identified as the key determinant of affinity with second generation TSPO radioligands [11C]DAA1106 and [11C]PBR28 [65]. Cerebral blood flow, which is reduced in AD, highly influences monoamine oxidase B (MAO-B) binding that seems to increase with age in almost all brain regions (with the exception of the cingulate gyrus) in healthy human subjects [62]. PET neuroinflammation imaging may, alternately, be more useful in monitoring the responsivity to anti-inflammatory therapies in AD.

#### PET imaging for amyloid load

Amyloid accumulation evidenced by florbetapir PET may be a potential marker of preclinical AD. Cognitively normal subjects with florbetapir uptake increase in brain were associated with worse globe cognitive performance [66]. [(11)C]PIB data showed expected differences among subjects of control, MCI and AD [67–69] and identified subjects with significant annual increases in amyloid load across the subject groups [67]. Longitudinal studies showed that PET Aβpositive subjects of cognitively normal, MCI and AD subjects had greater cognitive and global deterioration than Aβnegative subjects [70, 71] and Aβpositive subjects of MCI had higher risk for conversion to AD than Aβnegative subjects [71, 72]. Florbetapir PET measurements showed that 76 % AD, 38 % MCI, and 14 % cognitively normal subjects was amyloid positive [73]. The global cortex standardized uptake value ratio of Florbetapir could differentiate AD and MCI from healthy normal controls with high specificity and sensitivity [74]. Amyloid deposition has already slowed or ceased when dementia occurs [75]. Combination baseline sMRI and (18)F-florbetaben (FBB) uptake values did not improve predictive accuracy of MCI conversion to AD [76]. CSF Aβ42 did not always become abnormal prior to fibrillar Aβ accumulation early in the course of disease [77]. 2-(1-{6-[(2-fluorine 18-labeled fluoroethyl)methylamino]-2-napthyl}ethylidene) malononitrile ([(18)F]FDDNP) provides a measure of both amyloid and tau. [(18)F]FDDNP could predict future cognitive decline and conversion of MCI to AD [78].

#### PET radioligands of tau protein

In the amyloid cascade hypothesis, although tau pathology is considered secondary to Aβpathology, the post-mortem classification of AD cases into pathological subtypes with distinct clinical characteristics is determined by the localization and distribution of tau pathology in the brain [79, 80]. Abnormal burden of tau species can accurately predict disease severity and the rate of cognitive decline [79, 80]. The recent progress in tau protein ligands makes tau PET imaging a potential biomarker. Radioligands [18 F]-T807 and [18 F]-T808 used in human brain images with different pharmacokinetic characteristics were the most selective compounds for filamentous tau, and the level of selective binding with tau was 27-fold higher than that of Aβ [81, 82]. A phenyl/pyridinyl-butadienyl-benzothiazoles/benzothiazolium (PBB) showed the greatest specificity for tau in vivo PET imaging of tau transgenic mouse models, and [(11)C]PBB3 patterns were consistent with the spreading of tau pathology with AD progression and non-AD tauopathy corticobasal syndrome in a clinical PET study [83].

#### fMRI and SPECT

A set of medial prefrontal and temporo-parietal regions, such as the posterior cingulate and hippocampus, are referred to as the default-mode network (DMN), which is most active at rest and deactivated during cognitive tasks. Amyloid deposition in AD brains is most obvious in brain areas of the default network. Functional MRI showed that the DMN exhibits both reduced functional connectivity and impaired task-induced deactivation in AD, mild cognitive impairment (MCI) and pre-MCI [84–89].

Subjects with aMCI showed decreased DMN activity in memory function related brain regions, such as left medial temporal lobe before atrophy was detectable by sMRI [86]. Based on a multi-modal imaging approach, including FDG-PET and sMRI and diffusion-weighted MRI results, AD patients revealed decreased structural and functional connections, corresponding consistent reduction of metabolic activity and atrophy within DMN [90]. The disrupted connectivity in AD turns the high metabolic activity of DMN into hypometabolism [84]. A 2- to 3- year follow-up study revealed that functional connectivity indices could predict conversion of MCI to AD [91]. Education, a factor in cognitively demanding tasks, reduces AD risk by reducing neuronal activity and Aβ generation within the default network [92]. It was observed that in the earliest phase of MCI the individuals exhibited significantly greater hippocampal activation than controls even if two group without difference in hippocampal or entorhinal volumes [93]. Longitudinal fMRI in cognitively normal elderly participants reveals that subjects with the highest hippocampal activation at baseline and the greatest loss of hippocampal activation demonstrated more rapid cognition decline [94]. Compensatory efforts as a result of preclinical pathological changes in learning and memory tasks induce enhanced brain activity in some areas in the initial targets of AD, and these changes may precede the diagnosis of AD by 30 years [95]. Neuronal activity stimulates aerobic glycolysis and increases Aβ production and secretion into the interstitial fluid (ISF). The degree of Aβ aggregation and plaque deposition is directly proportional to ISF Aβ concentrations in vivo [96]. Factors including elevated endogenous neuronal activity may accelerate the Aβ deposition process. Cognitively normal ApoEε4 carriers show elevated resting-state activity in the default network and increased hippocampal activation in fMRI during a memory-encoding task compared to non-carriers [97]. Cerebral perfusion abnormalities were evident in AD progression. CBF was a more sensitive parameter than cerebral blood volume for perfusion normalities and appeared before the latter in the progression of AD [98]. A reduced CBF of the left posterior cingulated gyrus [99], bilateral prefrontal and frontal, and left parietal [100], and right parietal and hippocampal regions [101] evidenced by SPECT could predict the conversion of MCI to AD at least 2 years before clinical AD. But low parietal and medial temporal flow using SPECT demonstrated limited utility in predicting MCI conversion to AD [102]. Semi-quantitative circumferential-profile analysis of brain SPECT showed that AD patients have more significant reductions in the posterior temporo-parietal regions, and white matter VaD patients have greater reducutions in the frontal brain regions [103].

Combination the pattern of hypoperfusion and the severity of memory deficits could predict the risk of progression to AD in MCI subjects with a sensitivity and specificity [102, 104].

Regional CBF measured by Arterial spin-labeling (ASL) magnetic resonance showed that regional CBF of AD patients was significantly lower in both the bilateral frontal and temporal lobes, and the value of VaD patients was significantly in left frontal and temporal white matter [105]. SPECT imaging of regional benzodiazepine receptors (rBZR) can reflect neuronal integrity in the cerebral cortex. VaD and mixed AD/VaD dementia showed predominant reduction of regional CBF and rBZR in the frontal lobe, and AD in parietotemporal lobe. Furthermore, rBZR images of VaD and mixed dementia showed more extensive and severe defects than CBF images, and CBF images of AD showed more extensive defects than rBZR [106]. Although the heterogeneity index of the whole brain CBF on SPECT images was not significantly different between the AD and VaD groups, the herogeneity of CBF for AD and VaD was posterior and anterior-dominant respectively [107].

## Conclusion

The use in clinical practice of neuroimaging biomarkers of brain pathological processes could permit to perform an early diagnosis and to estimate the disease progression. Some neuroimaging-biomarkers have been widely used in clinical diagnosis of AD. The more neuroimaging biomarkers are still used for clinical studies. The use of neuroimaging biomarkers depended on the stage of disease progress. The proposed pathophysiological sequence contains Aβaccumulation, neuronal dysfunction (synaptic dysfunction, glial activation, tangle formation), brain atrophy due to neuronal death, and finally cognitive impairment [108].

Amyloid imaging is useful for differential diagnosis in early-onset dementia and clinical diagnosis of AD in noncarriers of ApoEε4 who are older than 70 years [5]. However, amyloid imaging may not be sufficient to make correct diagnosis in an individual [109]. PET with FDG, tau and other neurochemical tracers, fMRI and SPECT are used to measure the neuronal dysfunction. Finally, sMRI, advanced MRI techniques, such as DTI and MRS are used to show atrophy and hypoperfusion in cortex and white matter. The ordering and the sigmoidal-like time changes of imaging biomarkers are useful to detect early pathophysiological changes in preclinical stage; the extensive and severe impairment means greater AD-like pathology. Therefore, imaging biomarkers often are used to detect the comversion of preclinical AD and MCI to AD and predict outcomes of clinical intervention trials. Particularly, imaging biomarkers are helpful to differentiate AD dementia from VaD and other non-AD dementia. To find early different vascular pathological contribution to AD and VaD, an advanced dynamic contrast-enhanced MRI could quantify hippocampus blood brain barrier permeability in the living human brain; CA1 and dentate gyrus subdivisions showed obviously worsened in patients with mild cognitive impairment,which was correlated with injury to BBB-associated pericytes [110].

The combination of structural, functional neuroimaging and fluid biomarkers improved the accuracy of prediction [111]. With same diagnosis power, one should give preference to the less expensive, safer and less invasive techniques. However, some factors, including environmental factor, ApoE genetic variation and brain or cognitive reserve can affect or alter the cuves of these biomarkers.

## Ethics approval and consent to participate

Not applicable.

## Consent for publication

Not applicable.

## Availability of data and materials

Not applicable.

## Abbreviations

[(11)C]PIB: C-labeled Pittsburgh Compound-B
[(18)F]FDDNP: 2-(1-{6-[(2-fluorine 18-labeled fluoroethyl)methylamino]-2-napthyl}ethylidene) malononitrile
[11C]-DED: 11C-deuterium-L-deprenyl
AA: arachidonic acid
AD: Alzheimer’s disease
ApoE: apolipoprotein E
Aβ42: amyloid-β42
BPF: brain parenchymal fraction
CBF: cerebral blood flow
CNS: central nervous system
cPLA2: cytosolic phospholipase A2
CSF: cerebrospinal fluid
DMN: default-mode network
DTBZ: [11C]dihydrotetrabenazine
DTI: diffusion tensor imaging
FA: fractional anisotropy
FBB: (18)F-florbetaben
FDG: fludeoxyglucose
ISF: interstitial fluid
MAO-B: monoamine oxidase B
MCI: mild cognitive impairment
mMT: model-based magnetization transfer
MRI: magnetic resonance imaging
MTR: magnetization transfer ratio
NIAAA: National Institute on Aging-Alzheimer’s Association
PBB: phenyl/pyridinyl-butadienyl-benzothiazoles/benzothiazolium
PET: positron emission tomography
p-tau: phosphorylated tau
RA: radial diffusivity
rBZR: regional benzodiazepine receptors
rs6971: single nucleotide polymorphism
sMRI: structural MRI
SPECT: single-photon emission computed tomography
sPLA2: secretory phospholipase A2
STARDdem: Standards for the Reporting of Diagnostic accuracy studies in dementia
TSPO: translocator protein
VaD: vascular dementia
WMH: white matter hyperintensities
